# Reply to: Uncertainty and bias in Liggio et al. (2019) on CO_2_ emissions from oil sands operations

**DOI:** 10.1038/s41467-023-40819-4

**Published:** 2023-09-06

**Authors:** John Liggio, Shao-Meng Li

**Affiliations:** 1 Air Quality Research Division, Environment and Climate Change Canada, 4905 Dufferin St, Toronto, Ontario M3H 5T4 Canada; 2 College of Environmental Sciences and Engineering, Peking University, 100871 Beijing, China

**Keywords:** Climate-change mitigation, Attribution

**replying to** L. Fu & A. H. Legge *Nature Communications* 10.1038/s41467-023-40818-5 (2023)

In our paper^1^, we presented an aircraft measurement-based assessment of the CO_2_ emissions from the oil sands surface mining sector in Alberta, Canada, and demonstrated that overall CO_2_ emissions were 64% higher than reported by the industry. Subsequently, Fu and Legge provided several comments, indicating disagreements with the algorithm utilized (TERRA) and with the approaches used for upscaling emissions to annual values while also identifying a legitimate technical error and a typo in the scale of one figure. In our reply to their comments, we provide evidence that their arguments and comments were a result of incorrect scientific analysis and/or incorrect assumptions.

A starting point of their rebuttal, which appears in their title and throughout their discussion, is that the results of the work^1^ are biased. Bias, by definition, implies that the “truth,” or, in this case, the actual amount of CO_2_ emissions, is already known, which is not the case. For the oil sands sector, industries report bottom-up estimates, while we have provided aircraft-based top-down emissions estimates. It is not a surprise that the two methods provide different results, but neither method should be tagged as being biased because the truth is unknown. Similar comparisons between top-down and bottom-up approaches have been reported in many scientific publications, and there is now significant evidence that emissions from oil and gas operations globally are underreported in comparison to actual emissions. Further, a recent paper^2^, which used different upscaling methodologies and 15+ years of satellite measurements, shows the same discrepancy between measured and reported oil sands CO_2_ emissions as was shown in the previous work^1^. Regardless, we will address each of the arguments of Fu and Legge in the order in which they appear in their rebuttal.

## Issue 1. Technical errors

Fu and Legge assert that a single calculation error (not using the correct CO_2_/SO_2_ molecular weight ratio) invalidates the results from the emissions estimation algorithm (TERRA). This is incorrect. The molecular weight ratios of CO_2_/SO_2_ were not used in the calculation of the TERRA-derived emissions, nor were they used to directly validate TERRA-derived emissions. TERRA results were validated via direct comparison of SO_2_ to CEMS data as discussed previously^1,3^. The CO_2_/SO_2_ molecular weight ratio was used to provide a second and independent method for estimating/scaling up the CO_2_ emissions from stacks to compare with the results using the TERRA algorithm. In Liggio et al.^1^, we concluded that the two independent results were comparable within their respective uncertainties.

Further, it is important to realize that this molecular weight ratio error has no effect on Fig. 2 of the paper. Figure 2 is the primary figure in the paper, and it reflects the major conclusions of the paper (which are unrelated to the use of SO_2_ upscaling or the CO_2_/SO_2_ ratio). The error does affect Fig. 3. Specifically, in Fig. 3d, the green columns will be reduced by 31% so that the green column for SML will change from approximately 14 to 10, while the green column for SUN will change from approximately 7 to 5. The comparison that we were attempting to highlight in Fig. 3d was between the green (stack emissions based on ratios of molecular weight) and orange (stack emissions based on TERRA) columns. We concluded in the paper that they were comparable. With a 31% change (reduction) to the green column, they are certainly less comparable than they were for the SML facility. However, as we have already noted above, the overall CO_2_ emissions from the facilities (ground plus upgrading stack) are not largely impacted. Notably, recent satellite-based emissions data^4^ have demonstrated that the satellite-observed SO_2_ emissions for SML and SUN in 2013 (when the study was done) were approximately 30% higher than reported. This would cause the green bars in Fig. 3b to increase by 30% so that the results shown in the figure would essentially remain unchanged.

We have published a correction to the paper that identifies the molecular weight error and included a revised Fig. 3. However, as noted above, had we taken into account the results from McLinden et al.^4^, the figure would have remained essentially unchanged. Fu and Legge further point to differences between Figs. 2 and 3 as being symptomatic of an overall issue with the use of TERRA since the sum of estimates in Fig. 3 does not exactly add up to that of Fig. 2. The differences between Figs. 2 and 3 are expected, as the application of TERRA to different parts of a given flight will indeed result in slightly different estimates. This is accounted for in the error bars of both Figs. 2 and 3 and will not impact the main conclusion of the paper.

## Issue 2. Upscaling approach using CEMS NOx data

Fu and Legge suggest that there is an issue with the approach to upscaling of derived emissions. Their argument is that continuous emissions monitoring system (CEMS) NOx data should not be used as a basis for upscaling (similar to using SO_2_ data) because they are incompletely reported. They then use the argument of incomplete reporting to suggest that the previous approach^1^ was faulty and biased.

However, the original paper^1^ did not use CEMS NOx data in the manner they are suggesting. This is because CEMS only incorporates NOx from a few stacks for each facility while excluding some others and entirely excludes any mobile combustion emissions (of which there are many). That is precisely why we did not use a CO_2_ (measured)/NO_X_ (CEMS) emission factor approach, as Fu and Legge are suggesting. We agree with Fu and Legge that NOx CEMS data are likely to be incomplete. However, this is not relevant, as we are not using it directly to scale emissions of CO_2_. The majority of the analysis provided by Fu and Legge in their rebuttal uses a correlation between NOx as measured by CEMS and reported to inventories, versus CO_2_ as measured by the research aircraft. We believe that this comparison is invalid considering the limitations associated with CEMS NO_X_ reports noted above (i.e., not including all sources of NOx), while CO_2_ from the aircraft measures the complete CO_2_ from all sources within the facility.

Supplementary Figure S4b shows a correlation between NOx as measured by the aircraft versus CO_2_ as measured by the aircraft (measured at the same times and in the same plumes). The purpose of Supplementary Fig. S4b was to show that there is a high degree of correlation between facility integrated CO_2_ and NOx. It was not intended to be used as an upscaling approach, as Fu and Legge suggest. The relationship between measured CO_2_ and measured NOx was important since it was assumed that NOx is correlated with the production of synthetic crude oil (SCO). If NOx and CO_2_ are well correlated, then we can infer that CO_2_ is also correlated with the production of synthetic crude oil. Since synthetic crude oil production is reported monthly in inventories, we can scale flight measurements of CO_2_ emissions (taken over several hours) to monthly estimates of CO_2_ emissions and then correlate estimates of monthly CO_2_ emissions to monthly synthetic crude oil production. This is precisely how scaling was done in the paper, and this is well described in the text, including the noted uncertainties of the underlying relationship with NOx and SCO production.

Fu and Legge identified a typo in the units of the *x*-axis of Supplementary Fig. S4b (ppm listed, but it should have been ppb). The actual values for the data in the figure are correct (they are ppb), and we acknowledge the *x*-axis caption contains a simple typo, although it has no impact on any results reported.

While there may be different approaches to upscaling measurements, what Fu and Legge are suggesting is not credible because the TERRA emissions and the CEMS emissions are not fully comparing the same sources. Supplementary Figure S4b provides the groundwork for a more rigorous analysis of measured CO_2_ compared to measured NOx, followed by upscaling by total oil sands NO_X_ emissions (from all sources). This is beyond the scope of this paper.

## Issue 3. Perceived background procedure deficiencies and inconsistent application

Fu and Legge assert that the background subtraction performed by Liggio et al. was not consistently applied. They seem to be confusing “*constant extrapolation*” below the lowest flight altitude with “background subtraction” in general. The constant extrapolation was used below the lowest flight track to account for any CO_2_ that was not directly measured. Previous work has already demonstrated that this extrapolation is the largest source of uncertainty in TERRA, but the differences between extrapolation methods are small and, nonetheless, are included in the uncertainty estimates. Background CO_2_ was determined differently and was not a constant extrapolation. The background levels of CO_2_ for any given flight did exhibit minimal variability (0.5–3 ppm, as noted in the paper) relative to the enhancement caused by oil sands emissions (>80 ppm in some flights). Further, the premise of the developed background subtraction algorithm used in the paper was that it was free of bias and, in fact, applied consistently for every flight, contrary to their assertion. In addition, the impact of the application of a conservative varying of the background was conducted (±1 σ) so that the impact of the background subtraction on the final emissions could be included in the uncertainty analysis. It has already been shown in the paper that significantly varying the baseline CO_2_ had only a small effect on the uncertainty of the final results^1^.

Additionally, Fu and Legge chose data from a specific flight (F6) and then suggested that it shows inconsistent background levels for CO_2_, issues with meteorology, and emission below the lowest flight track. Their argument in this regard is unclear, as the plumes for SUN and SML were detected at different downwind location, and on different screens, such that they have minimal influence on each other. Further, the enhancement in CO_2_ is well above any nearby background CO_2_ determined. Fu and Legge also state that 2/3 of the SML emissions within this flight (F6) are below the lowest flight track. This is of no consequence, as the SI figure shows concentrations, not emissions through each screen, which are dependent on wind speeds (i.e., 2/3 of the concentration may remain a small emission flux). Overall, below-flight track concentrations/emissions are already a well-known uncertainty in TERRA (and all other aircraft mass balance methods), and it is clearly accounted for and described in our paper. As such, their text of section 3 of their “*Matters arising*” does not provide any additional insight into this well-known issue.

Fu and Legge also suggest that pre-buildup (i.e., “storage and release events”) of emissions adversely impacted the flights in this work. This is a significant oversimplification. The process of “pre-buildup” has recently been investigated^5^. That paper demonstrated that the contribution of “storage and release” events is likely to be minimal (<2–17%) for certain meteorological conditions and within the overall uncertainty of the TERRA approach. Fu and Legge suggest that emissions from SML adversely impacted the emissions for flights F14 and F7 and cite the Fathi et al. paper as a reason to reject this flight, but they provide no supporting evidence. The Fathi et al. paper^5^ is a modeling study using model meteorology that is designed to demonstrate the types of conditions required for such events to occur. It does not imply they have actually occurred. Regardless, emissions on those days are not significantly different than emissions on other days for the same facilities, and hence there is no clear justification for removing any flights. Regardless, the exclusion of one flight (with a similar emission value) does not impact the results of this work.

Any top-down measurement of emissions comes with a number of associated uncertainties which is well established in the literature on this topic. In our paper, we have specifically discussed several quantifiable and unquantifiable uncertainties, including those associated with: (1) emissions below the lowest flight track, (2) background levels of CO_2_, (3) the proportionality of NOx to oil production, (4) the variability across flights, (5) use of scaling factors, (6) oil production volumes, (7) the inclusion of methane into the GHG estimates, (8) the impact of local vehicle emissions, and (9) emission seasonality. These uncertainties, and various others, are already described in the original paper under “Other considerations and assumptions” in “Methods.” While we did not mention meteorology at the time, we acknowledge that the impact of meteorological conditions is an important factor in designing aircraft studies for the purpose of emissions estimation. Fu and Legge also suggest that the TERRA code was not available to them to verify our results. This is incorrect, as it is clearly stated in the published paper, although it has not been requested by Fu et al.

Overall, the rebuttal of Fu and Legge is problematic, as many of their assumptions are not supported, and they provide numbers that are not backed up by calculations or references, and some information is taken out of context. The one legitimate item that they identified is related to a calculation error that affects Fig. 3d in Liggio et al.^1^, and for which we have already published a correction.

## Data Availability

No new data was used for this reply. The authors declare that the data supporting the findings of the original study are available within the original paper and its Supplementary Information files. Should any raw data files be needed in another format, they are available from the corresponding author upon reasonable request.
